# Does diet or macronutrients intake drive the structure and function of gut microbiota?

**DOI:** 10.3389/fmicb.2023.1126189

**Published:** 2023-02-13

**Authors:** Yuhang Li, Yujie Yan, Hengguang Fu, Shiyu Jin, Shujun He, Zi Wang, Guixin Dong, Baoguo Li, Songtao Guo

**Affiliations:** ^1^Shaanxi Key Laboratory for Animal Conservation, College of Life Sciences, Northwest University, Xi’an, Shaanxi, China; ^2^Guangdong Chimelong Group Co., Ltd., Guangzhou, China; ^3^Guangdong South China Rare Wild Animal Species Conservation Center, Zhuhai, China; ^4^Shaanxi Institute of Zoology, Xi’an, China; ^5^Northwest Institute of Plateau Biology, Chinese Academy of Sciences, Xining, Qinghai, China

**Keywords:** 16S rRNA gene sequencing, metagenomic, metabolic functions, gut microbiota, wild golden snub-nosed monkey, seasonal variations

## Abstract

Shift of ingestive behavior is an important strategy for animals to adapt to change of the environment. We knew that shifts in animal dietary habits lead to changes in the structure of the gut microbiota, but we are not sure about if changes in the composition and function of the gut microbiota respond to changes in the nutrient intake or food items. To investigate how animal feeding strategies affect nutrient intakes and thus alter the composition and digestion function of gut microbiota, we selected a group of wild primate group for the study. We quantified their diet and macronutrients intake in four seasons of a year, and instant fecal samples were analyzed by high-throughput sequencing of 16S rRNA and metagenomics. These results demonstrated that the main reason that causes seasonal shifts of gut microbiota is the macronutrient variation induced by seasonal dietary differences. Gut microbes can help to compensate for insufficient macronutrients intake of the host through microbial metabolic functions. This study contributes to a deeper understanding of the causes of seasonal variation in host-microbial variation in wild primates.

## 1. Introduction

What factors cause or have interaction with gut microbiota is a key and hot issue in animal evolutionary adaptation and original of human diet health. It is reported that the composition and structure of animal gut microbiota changes with host diet, which is shown in macroscopic indicators such as diversity of the microbes (Hooper et al., 2012; Morgan et al., 2012; Markle et al., 2013; Amato et al., 2019). Moreover, changes of gut microbiota show seasonal fluctuation in wildlife and humans, and literatures also widely indicated that seasonal dietary changes lead to the reconfiguration of gut microbiota of hosts, or at least both aspects have strong interaction. For example, seasonal cycling in the gut microbes following the dietary fluctuation has been reported in the Hadza hunter-gatherers in Tanzania (Smits et al., 2017), the western lowland gorillas and chimpanzees (Hicks et al., 2018), and red squirrels (Ren et al., 2017).

While, recent studies have shown that diets of invertebrates and vertebrates may be determined by the nutrient components in foods (Ruohonen et al., 2007; Simpson and Raubenheimer, 2012). The intake of each macronutrient should be stable no matter whether an animal is a vegetarian, carnivore, or omnivore and how complex its food composition is (Raubenheimer et al., 2009, 2015; Machovsky-Capuska et al., 2016). Non-human primates can take in stable proportions of macronutrients from foods with complex and diverse components, which suggests that they have stable requirements for three macronutrients. The nutrient intakes of non-human primates are influenced by both the type and the amount of food consumed. Studies on folivorous primates including species of the subfamily Colobus (Chapman et al., 2003) and the subfamily Indriidae (*Indri*) (Junge et al., 2009; Fleming and John Kress, 2011) show huge variation in macronutrient composition in their foods. Meanwhile, some species such as Mountain gorillas (*Gorilla Beringei*) can maintain a constant non-protein intake during the period when non-protein nutrients from fruits are scarce by consuming an excessive amount of leaves (Rothman et al., 2011).

As the diet may co-evolve with gut microbiota among different animals, the notion that diet variation can influence gut microbiome composition and structure has been confirmed at the taxonomic level of family (Ley et al., 2008). However, within genera, or ranks below genera, species from the same taxa may vary greatly in diet (Guo et al., 2007). For example, chimpanzees (*Pan troglodytes*) are considered frugivorous primates, but they feed heavily on leaves and even prey on other animals during the fruitless season (Rothman et al., 2007). The dietary variability makes it difficult to explain the changes in the composition and structure of gut microbes. So far, it has been found that such changes are closely related to the macronutrients consumed on the study of captive animals (Grześkowiak et al., 2015). However, due to the difficulty in quantifying the nutrient intakes in the field and the scarcity of such studies on wild animals, especially endangered wild mammals, we are hampered to figure out the mechanisms of gut microbiota-host co-evolution in many wild species. Therefore, this study aims to investigate how whether diet or nutrients intake affect the structure and composition of gut microbiota and the interaction among these three aspects. This will reveal the mechanisms behind the seasonal shifts in diet in animals that rely heavily on gut microbiota for digestion.

Golden snub-nosed monkeys (*Rhinopithecus roxellana*) belong to the genus *Rhinopithecus* in the subfamily Colobinae, and their habitats vary seasonally (Hou et al., 2018). Our previous research on their diet based on time ratio reveals that wild golden snub-nosed monkeys have seasonal diet variation (Guo et al., 2007). Recent studies show that their feeding strategy stabilizes protein intakes and balances energy requirements by regulating carbohydrate and lipid intakes (Hou et al., 2021). Since golden snub-nosed monkeys live in an environment with complex foods and are capable of maintaining a stable amount of macronutrient *via* various feeding strategies, they are an excellent model for studying the interactions between food consumed, nutrient intakes, and gut microbiota. In view of the reasons above, we propose the following research questions. (1) Do seasonal changes in food types lead to changes in the composition and structure of gut microbiota? and (2) Do seasonal variations in nutrient intakes lead to seasonal variations in gut microbiota composition? (3) Are there any seasonal variations in gut microbial gene function? Does the gene function correlate with seasonal variations in dietary and nutrient intakes?

## 2. Materials and methods

### 2.1. Data collection

Our observation site was in Guanyin Mountain National Nature Reserve (107°51′-108°01′E，33°35′–33°45′N,135.34 km^2^) on the southern slope of Qinling Mountains, which locates at the northwest of Fuping County, Shaanxi Province, China (Supplementary Figure S1). This region experiences the classic and distinct four seasons throughout the year. The seasons are divided according to climate: spring is from March to May, summer is from June to August, autumn is from September to November, and winter is from December to February (Guo et al., 2007, 2018). We collected feeding data of four season groups (i.e., spring, summer, autumn, and winter). For each season, we chose a month with typical phenological characters, that is, March (spring), June (summer), October (autumn), and December (winter) for data collection.

Our study group of golden snub-nosed monkeys had 78 individuals, all haven been habitualized to the presence of researchers. Adult and juvenile individuals haven been identified by us. Because we needed to collect quantitative observation data, the natural feeding space of the target animals was narrowed. To prevent that their total energy intakes being reduced due to this condition and thus impacting their health, we referred to our previous experience to provision foods (Hou et al., 2021). We provisioned 5 kg of maize grains twice daily at 10 a.m. and 3 p.m. as supplementary food for the group. Maize grains were spread evenly in the feeding grounds.

We randomly chose one individual per day and conducted continuous observations of the focal animal from dawn to dark to record its feeding data. During the observation session, the distance between the observer and the subject was less than 5 m. We recorded the type, quantity, and predefined units of the food and the amount of time feeding (Hou et al., 2018). After the focal individual completed feeding, leftover foods were collected as food samples. All samples were labeled with the information of the collection time, type, and size. Then, they were immediately frozen in liquid nitrogen and sent to the laboratory for storage before the analysis of their nutrient components. We also collected same-day fecal samples for high-throughput sequencing. After the focal individual defecated, we immediately collected the feces with sterile cotton swabs and sterile toothpicks. The sample was then stored in 2 mL centrifuge tubes and frozen in liquid nitrogen before being delivered for testing.

### 2.2. Nutrient analysis

We used the standard techniques to collect the food samples (Rothman et al., 2012), analyzed the foods nutrients, and calculated the energy values (Jung, 1995) with the same methods used in our previous studies (Guo et al., 2018; Hou et al., 2018). The macronutrients of each food were analyzed for lipid, water-soluble carbohydrate (WSC), starch, neutral detergent fiber (NDF), acid detergent fiber (ADF), acid detergent lignin (ADL), available protein (AP), and ash content. Available proteins are determined by the standard Kjeldahl method (using BUCHI, K-360).

To calculate the daily nutrient intake (DNI), we multiplied the nutrient content of each food item by the recorded amount of that item consumed, then summed these values for all items consumed by that individual on that day. We also calculated the rate of nutrient ingestion per hour (NIH) for each individual by dividing the amount of nutrient ingested by the amount of hours the focal animal was observed. The rate was multiplied by the sunshine duration to estimate the total daily intake (TDNI; Rothman et al., 2008). We lured each monkey with a small portion of food and led it onto a platform scale (accuracy, 0.02 kg; EM-60KAL, A&D, Japan) to record their weight when the readings were stable (Hou et al., 2021). To standardize weight differences between individuals, the calculation was divided by the individual’s estimated metabolic body mass (MBM = M^0.762^, where M is the body weight in kg).

### 2.3. DNA extraction and 16S rRNA gene sequencing

The microbial DNA (with a total mass of 1.2–10.0 ng) was isolated from each fecal sample using the MOBIO Pow erSoil DNA Isolation Kit and was quantified with NanoDrop One (Thermo Fisher Scientific, Waltham, MA, United States). The V4 regions of the DNA genes were amplified by using the specific primer 515F (5′-GTGCCAGCMGCCGCGGTAA-3′) and 806R (5′-GGACTACHVGGGTW TCTAAT-3′) with 12 bp barcode. Primers were synthesized by Invitrogen (Invitrogen, Carlsbad, CA, United States). The PCR instrument was Bio-Rad S1000 (Bio-Rad Laboratory, CA, United States). The length and concentration of the PCR product were detected by 1% agarose gel electrophoresis. PCR products with bright main strip between were mixed in equidensity ratios according to the GeneTools Analysis Software (Version 4.03.05.0, SynGene). Then, mixture of PCR products was purified with E.Z.N.A. Gel Extraction Kit (Omega, United States). Sequencing libraries were generated using NEBNext^®^ Ultra^™^ II DNA Library Prep Kit for Illumina^®^ (New England Biolabs, MA, United States) following the manufacturer’s recommendations and index codes were added. The library quality was assessed on the Qubit@ 2.0 Fluorometer (Thermo Fisher Scientific, MA, United States). At last, the library was sequenced on an Illumina Nova6000 platform and 250 bp paired-end reads were generated (Guangdong Magigene Biotechnology Co., Ltd. Guangzhou, China).

### 2.4. Metagenomic sequencing and gene catalog construction

The sequencing library was created using NEBNext Ultra DNA Library Prep Kit for Illumina (New England Biolabs, Beverly, MA, United States) and indexes were added to attribute sequences to each sample. The DNA sample was fragmented by sonication to a size of 300 bp. DNA fragments were polished at the extremities and were attached to the full-length adapter for Illumina sequencing with further PCR amplification. The library was analyzed for size distribution by Agilent2100 Bioanalyzer (Agilent, United States), and then was sequenced by Illumina HiSeq 2500 platform in Magigene Co., Ltd. (Shenzhen, China).

Quality control was conducted by Trimmomatic (Version 0.38). The reads aligned to the NCBI non-redundant (NR) database were removed with MEGAHIT (Version 1.05). The remaining high-quality reads were used for further analysis. The assembly of reads was conducted using MEGAHIT *de novo*. For each sample, a series of k-mer (substrings of length k) values (49–87) were used and the optimal one with the longest N50 value was chosen for the remaining scaffolds. The clean data were mapped against scaffolds using MEGAHIT. Unused reads from each sample were assembled using the same parameters.

Genes (minimum length of 100 nucleotides) were predicted on scaftigs longer than 500 bp using Prodigal (Version 2.6.3). Then, a non-redundant gene catalog was constructed with Linclust (Version 2.0) using a sequence identity cut-off of 0.9. To determine the abundance of genes, reads were realigned to the gene catalog with BBMap (Version 37.68). Only genes with 2 mapped reads no less than 2 were considered exist in a sample. The abundance of genes was calculated by counting the number of reads and normalizing by gene length. Genes were then searched in Kyoto Encyclopedia of Genes and Genomes (KEGG) database (Kanehisa and Goto, 2000) for annotation.

### 2.5. Statistical analysis

Kruskal-Wallis test with Bonferroni correction for multiple post-hoc pairwise comparison was used to compare the difference of available protein, fat, and carbohydrate in four seasons. Permutational Multivariate Analysis of Variance (PERMANOVA) was used to compare the difference of Shannon index and Chao1 index. The Analysis of Similarities (ANOSM) was used to compare the result of Principal co-ordinate analysis (PCoA). The Mantel test was used to compare the correlation between food groups and the gut microbial composition in each season. Pearson’s correlation coefficients were calculated to analyze correlations between weighted gene co-expression network analysis (WGCNA) module groupings and traits. Data visualization was performed by R4.1.3 and Cytoscape 3.8.2.

## 3. Results

### 3.1. Seasonal diets

In this study, feeding data of 96 days across 4 months (25 days in spring, 24 days in summer, 24 days in autumn, and 23 days in winter) were collected from the target group. It was observed that the natural foods consumed by wild golden snub-nosed monkeys consisted of 24 plant species belonging to 16 families. Six items of plant including barks, seeds, buds, brunches, leaves, and stems have been observed to be consumed. Throughout the year, the proportion of each plant item consumed by wild snub-nosed monkeys was 33.43% for bark, 3.09% for seed, 1.33% for bud, 3.25% for brunch, 0.17% for stem, and 58.72% for leaf. However, there were huge differences in the amount of the plant items being consumed across four seasons. Herbaceous stems were only taken in spring with a small quantity. Seeds were taken mainly in spring and autumn. Leaves were taken throughout the year. Buds, barks, and brunches were the main food in autumn and winter when leaves become scarce, especially in winter (Supplementary Figure S2A).

In addition, differences have also been found in the plant species consumed between seasons—*Photinia beauverdiana*, *Acer davidii*, *Dendrobenthamia japonic*a, *Kerria japonica*, *Ulmus macrocarpa*, *Quercus aliena*, *Acer mono Maxim*, and *Lonicera japonica* were mainly taken in spring; *Cerasus clarofolia*, *Ailanthus altissima*, *Juglans mandshurica*, and *Spiraea blumei* were mainly taken in summer; *Rubus pungens*, *Arachis hypogaea*, *Quercus mongolica*, *Pinus bungeana*, *Lonicera hispida*, and *Carpinus cordata* were mainly taken in autumn; and *Litsea pungens*, *Quercus dolicholepis*, *Fargesia qinlingensis*, *Bothrocaryum controversum*, *Glechoma longituba*, *Litchi chinensis*, and *Callicarpa nudiflora* were mainly taken in winter (Supplementary Figure S2B). The results of PCA on seasonal percentage data of food mass for 24 plant species and 6 plant items showed that there were significant differences in the plant species consumed by golden snub-nosed monkeys between any two seasons (PERMANOVA, *p* < 0.05, Table 1). Also, there were significant differences in plant items consumed between any two seasons except for spring and summer (PERMANOVA, *p* < 0.05, Table 2).

**Table 1:** Statistical analysis (PERMANOVA) of food mass seasonal difference of plant species consumed by wild golden snub-nosed monkeys.

Group	*F*-Model	*R* ^2^	Value of *p*	*p-*adjusted
Spring vs. Summer	6.979	0.145	0.001	0.0012
Spring vs. Autumn	7.155	0.143	0.001	0.0012
Spring vs. Winter	10.429	0.192	0.001	0.0012
Summer vs. Autumn	10.373	0.206	0.001	0.0012
Summer vs. Winter	15.486	0.274	0.001	0.0012
Autumn vs. Winter	3.787	0.081	0.003	0.0030

**Table 2:** Statistical analysis (PERMANOVA) of food mass seasonal difference of plant items consumed by wild golden snub-nosed monkeys (PERMANOVA).

Group	*F*-Model	*R* ^2^	Value of *p*	*p*-adjusted
Spring vs. Summer	1.548	0.036	0.184	0.1840
Spring vs. Autumn	8.362	0.163	0.001	0.0015
Spring vs. Winter	14.559	0.249	0.001	0.0015
Summer vs. Autumn	8.275	0.171	0.001	0.0015
Summer vs. Winter	12.679	0.236	0.001	0.0015
Autumn vs. Winter	3.451	0.074	0.005	0.0060

### 3.2. Nutritional properties

**Figure 1:**
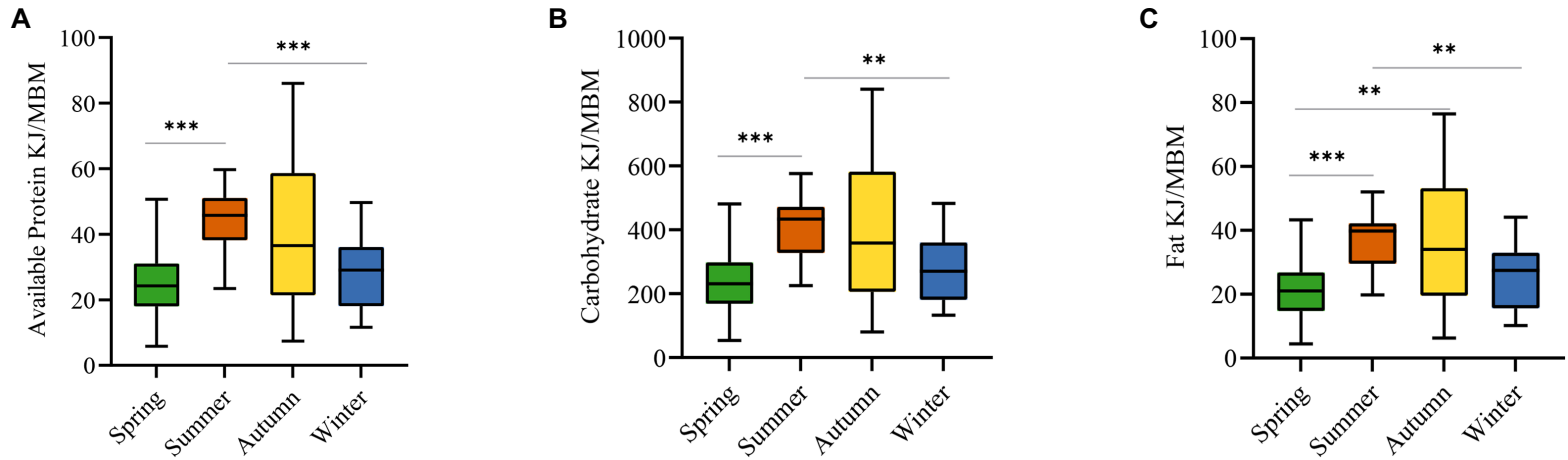
Comparison of macronutrients among four seasons (we measured the energy of metabolic body mass (KJ/MBM) provided by macronutrients). **(A)** The comparison of available protein intake. **(B)** The comparison of carbohydrate intake. **(C)** The comparison of fat intake. **p < 0.01, ***p < 0.001. (Kruskal-Wallis test).

We collected 55 types of food from 24 plant species and evaluated their nutritional properties. We measured the energy of metabolic body mass (KJ/MBM) provided by macronutrients, which showed that spring food intake provides 25.93 ± 11.04 kJ/MBM (M ± SE) of available protein, 22.41 ± 10.55 kJ/MBM of fats, and 245.83 ± 106.47 kJ/MBM of carbohydrates; summer food intake provides 43.80 ± 9.72 kJ/MBM of available protein, 36.88 ± 9.21 kJ/MBM of fats, and 411.19 ± 96.55 kJ/MBM of carbohydrates; autumn food intake provides 39.83 ± 22.26 kJ/MBM of available protein, 37.17 kJ/MBM of fats, and 394.68 ± 219.21 kJ/MBM of carbohydrates; winter food intake provides 28.17 ± 10.56 kJ/MBM of available protein, 24.76 ± 9.68 kJ/MBM of fats provides, and carbohydrates provided 278.15 ± 100.48 kJ/MBM. Statistical analysis of available proteins, carbohydrates, and fats provided by foods in four seasons found that they were all significantly different between spring vs. summer and summer vs. winter groups, and that fats also differed in spring vs. autumn group (Figure 1).

We also divided the sources of macronutrients into natural foods and artificial foods. For natural foods, the energy provided by available proteins decreases in the sequence of summer, winter, autumn, and spring, while the energy provided by carbohydrates and fats increases in the sequence of spring, summer, autumn, and winter. For artificial foods, available proteins, carbohydrates, and fats presented a uniform seasonal pattern throughout the year with energy values decreasing from summer to autumn to winter and to spring (Supplementary Figure S3).

### 3.3. Microbial compositions

**Figure 2:**
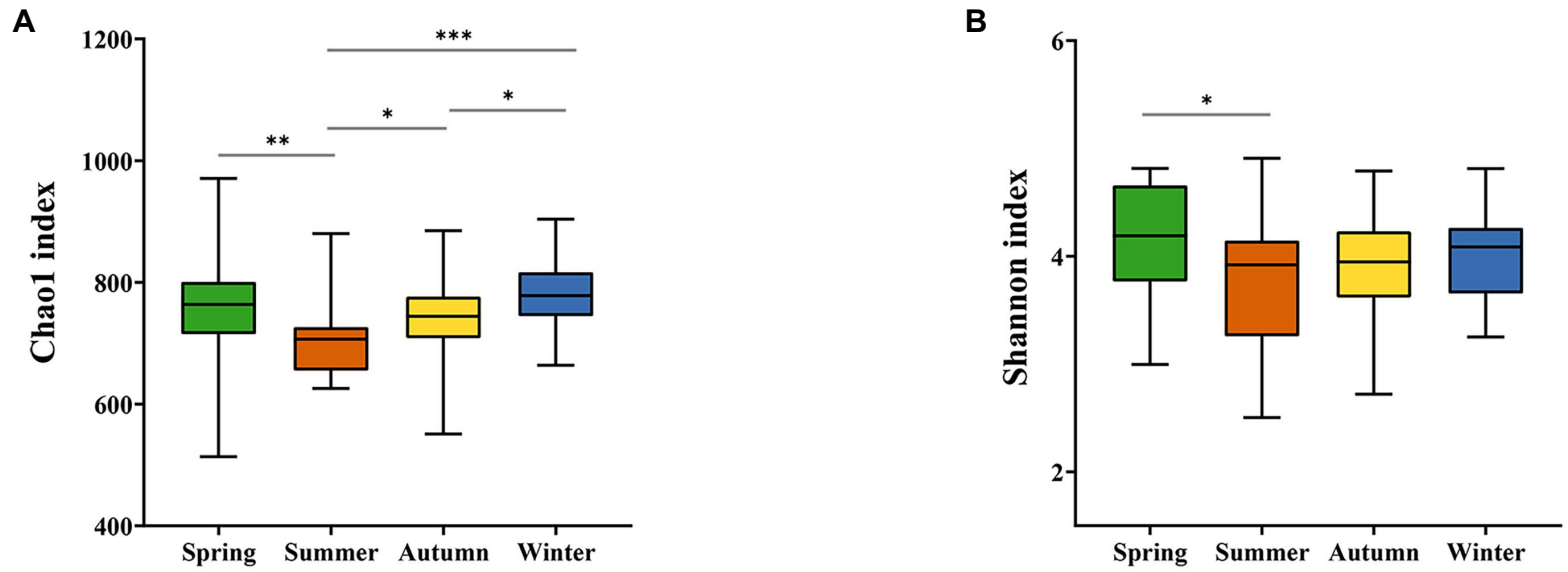
Alpha-diversity of gut microbiota. The Chao1 indexes fluctuate significantly between seasons **(A)**, while the Shannon indexes vary only in spring and summer and are stable for the rest **(B)**. *p < 0.05, **p < 0.01, ***p < 0.001 (Kruskal-Wallis test).

**Figure 3:**
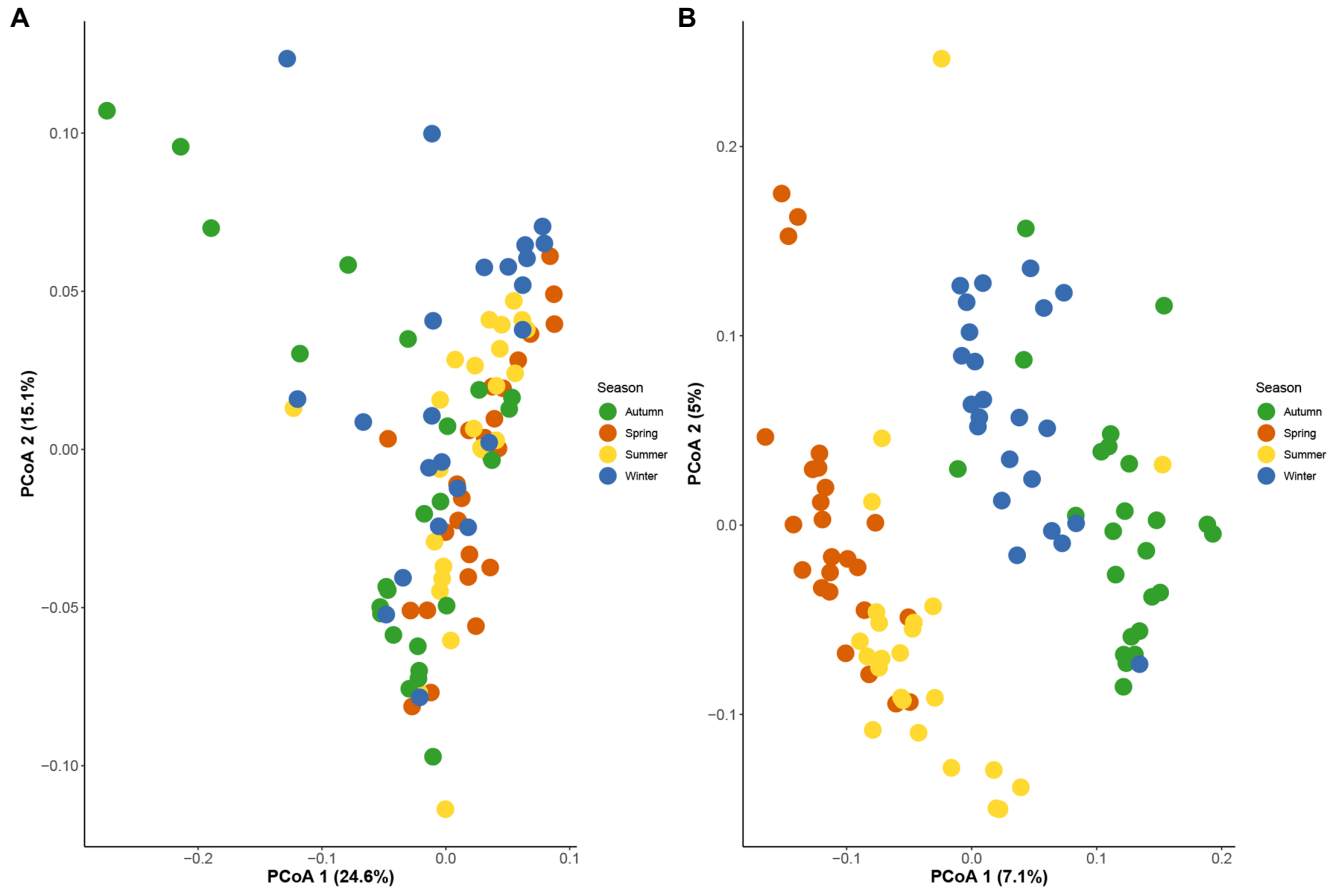
Principal co-ordinate analysis based on weighted UniFrac distances **(A)** and unweighted UniFrac distances **(B)** between all seasons showed significant difference in structure of seasonal gut microbial composition.

The 16S rRNA gene sequencing of fecal samples revealed that the observed species and the two alpha-diversity indexes reflecting species richness and diversity (Chao1 index and Shannon index, respectively) showed a decreasing in the order of spring, winter, autumn, and summer. That is, the richness of gut microbiota and the diversity of community in golden snub-nosed monkeys were highest in spring and lowest in summer during all seasons. The Chao1 index was significantly different between spring vs. summer, summer vs. autumn, summer vs. winter, and autumn vs. winter (Kruskal-Wallis test, *p* < 0.05, Figure 2A), while the Shannon index was only significantly different between spring vs. summer (Kruskal-Wallis test, *p* < 0.05, Figure 2B). Principal co-ordinate analysis (PCoA) based on weighted UniFrac distances (Figure 3A) and unweighted UniFrac distances (Figure 3B) of OTUs showed divergence between the groups of different seasons along the first and second principal components. Analysis of Similarities (ANOSM) showed that *p* values of all groups were less than 0.05, indicating a significant difference between the gut microbiota of four seasons.

**Figure 4:**
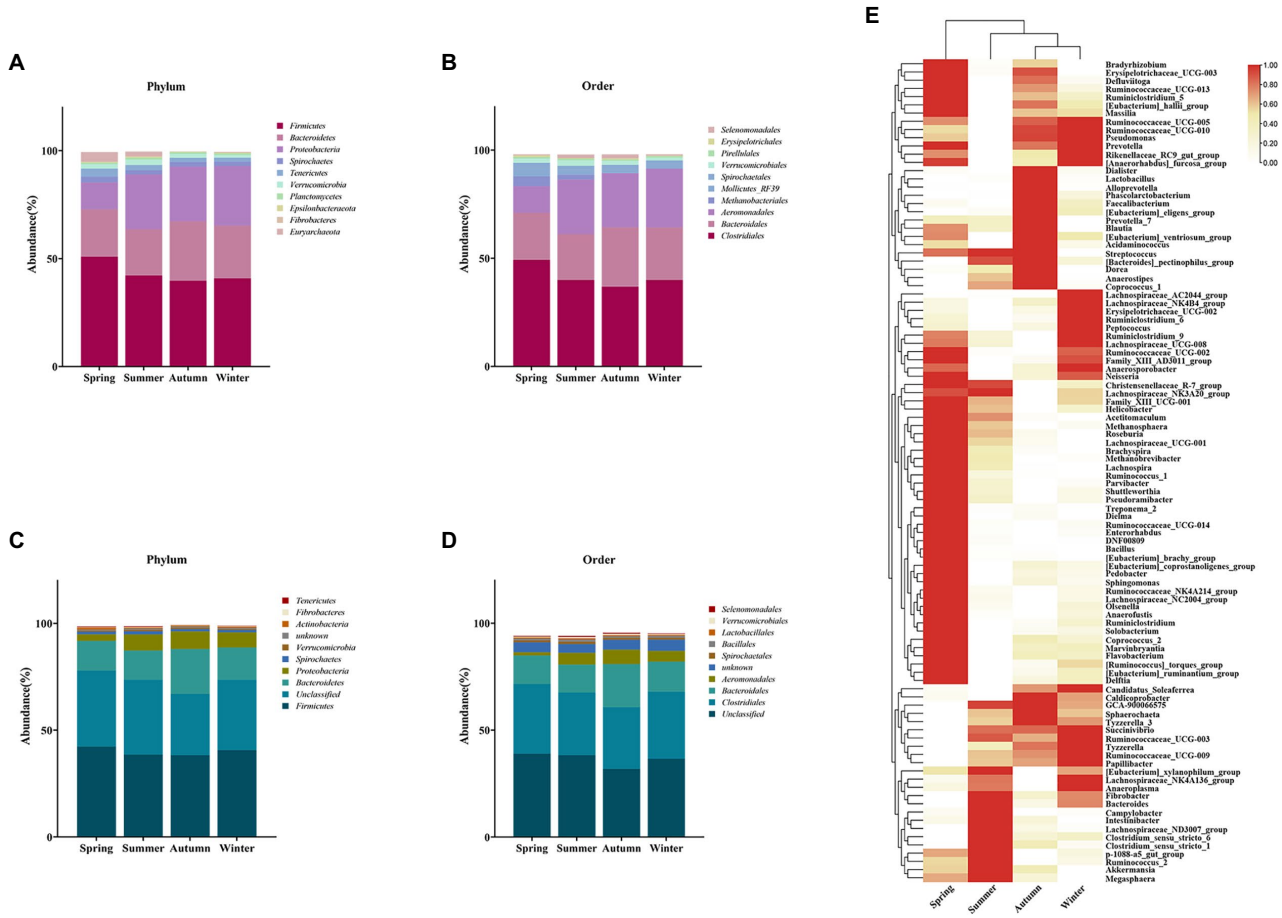
Analysis of seasonal differences in dominant bacterial populations. Relative abundance of dominant phylum **(A)** and order **(B)** in four seasons based on 16S rRNA gene pools. In contrast, macrogenome annotation of dominant bacteria at the level of phylum **(C)** and order **(D)**. Heat map of proportions at the genus level **(E)**, the darker the color, the higher the proportion of this genus present in the season compared to other seasons.

To further investigate seasonal differences in gut microbiota, species composition was analyzed. Species annotation of the 16S rRNA sequencing showed that most OTUs could be taxonomically assigned to the phylum (96%) and order (92%) level, but assignments decreased substantially at the genus (38%) level. A total of 38 phyla were annotated, of which the top 10 identifiable dominant phylum including *Firmicutes*, *Bacteroidetes*, *Proteobacteria*, *Spirochaetes*, *Tenericutes*, *Verrucomicrobia*, *Planctomycetes*, *Epsilonbacteraeota*, *Fibrobacteres*, and *Euryarchaeota* accounted for 99% of the total abundance ratio. These formed the core gut microbiota of golden snub-nosed monkeys. When considering them in different seasons, they showed seasonal differences in abundance, with *Firmicutes* and *Euryarchaeota* being most abundant in spring and *Proteobacteria* being least abundant in spring compared to other seasons (Figure 4A). The ratio of *Firmicutes* to *Bacteroidetes* (F/B) was highest in spring and lowest in autumn, suggesting a seasonal variation in the capacity of energy absorption by gut microbiota (Supplementary Figure S4).

A total of 140 orders were annotated. The dominant identifiable bacteria were *Clostridiales*, *Bacteroidales*, *Aeromonadales*, *Methanobacteriales*, *Mollicutes_RF39*, *Spirochaetales*, *Verrucomicrobiales*, *Pirellulales*, *Erysipelotrichales*, and *Selenomonadales* (Figure 4B). It is noteworthy that the abundance of the *Aeromonadales* was much lower in spring than in other seasons. Meanwhile, *Methanobacteriales*, which are associated with methane production, were observed to have abundance much higher in spring and summer than in autumn and winter. They had a particular high abundance in spring. Metagenomic analysis showed that several bacterial taxa with high abundance at the phylum and order levels were consistent with the 16S rRNA study (Figures 4C,D).

At the genus level, there were 352 taxa annotated and the top 100 genera covered nearly 99.9% of the total abundance. The study of these 100 genera found that the gut microbes of golden snub-nosed monkeys were mostly related to hindgut fermentation in ruminant animals. These genera include those that can degrade complex polysaccharides such as *Methanobrevibacter*, *Methanosphaera*, *prevotella_7*, *Roseburia*, *Ruminococcaceae_UGG-014*, *Treponema_2*, *Clostridium*, those that can produce hydrogen efficiently such as *christensenellaceae_R-7_group*, and those play roles in lipid metabolism such as *[Eubacterium]_coprostanoligenes_group*, *Blautia*, *Dorea*, *lactobacillus*, *Dialister*, and *Phascolarctobacterium* (Figure 4E).

### 3.4. Gene function prediction of gut microbiota

We performed KEGG annotation using metagenome data to find out the main functions of the gut microbiota in golden snub-nosed monkeys. According to the function prediction based on the KEGG database, we identified 395 metabolic pathways. Among these pathways, gut microbes were mainly involved in the nucleotide metabolism, carbohydrates metabolism, glycans metabolism and biosynthesis, amino acids metabolism, energy metabolism, lipids metabolism, terpenoids and polyketides metabolism, as well as cofactors and vitamins metabolism. Moreover, some functions annotated concerning macronutrients showed relatively high abundance such as glycolysis/gluconeogenesis, pyruvate metabolism, starch and sucrose metabolism, pentose phosphate pathway in carbohydrate metabolism and glycerophospholipid metabolism, glycerolipid metabolism, and fatty acid synthesis in lipid metabolism.

**Figure 5:**
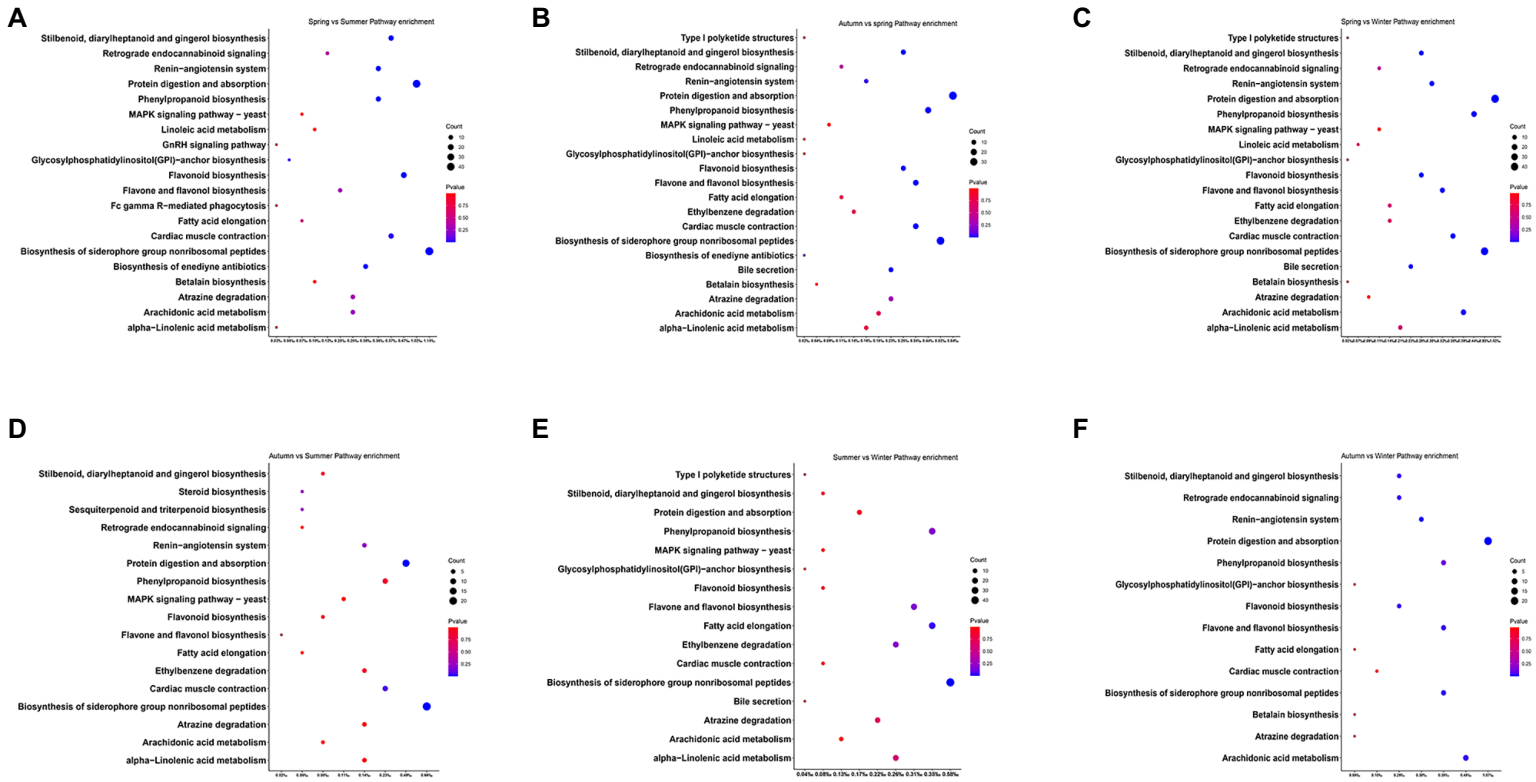
Analysis of KEGG annotation in groups with significant differences in macronutrient intakes to predict the function of gut microbial, including spring vs. summer group **(A)**, autumn vs. spring group **(B)**, spring vs. winter **(C)**, autumn vs. summer **(D)**, summer vs. winter group **(E)**, and autumn vs. winter **(F)**.

Inter-seasonal KEGG enrichment analysis demonstrated that there were 288, 210, 237, 98, 71, and 78 differentially expressed genes and were enriched in 20, 21, 20, 17, 16, and 14 KEGG pathways in spring vs. summer, spring vs. autumn, spring vs. winter, summer vs. autumn, summer vs. winter, and autumn vs. winter groups, respectively. The seasonal function variation of these differentially expressed genes can be presented in terms of metabolism, organic systems, and environmental information processing. Specifically, the enriched pathways were similar in all season groups. These pathways included biosynthesis of siderophore group nonribosomal peptides (ko01053); protein digestion and absorption (ko04974); flavonoid biosynthesis (ko00941); stilbenoid, diarylheptanoid, and gingerol biosynthesis (ko00945); cardiac muscle contraction (ko04260), phenylpropanoid biosynthesis (ko00940); arachidonic acid metabolism (ko00590); atrazine degradation (ko00791); flavone and flavonol biosynthesis (ko00944); linoleic acid metabolism (ko00591), fatty acid elongation (ko00062); alpha-Linolenic acid metabolism (ko00592); and bile secretion (ko04976) (Figure 5).

### 3.5. Correlation between gut microbiota and food types

**Figure 6:**
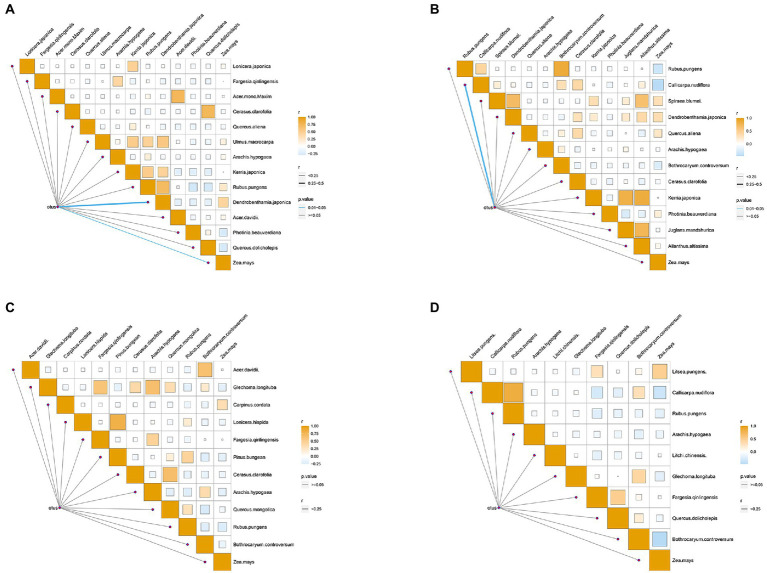
Heat map of the correlation between food groups and gut microbes of spring **(A)**, summer **(B)**, autumn **(C)**, winter **(D)**. Darker color refers to that r value is closer to 1; thicker line refers to higher *r* value between food groups and gut microbes. The color of the line segment shows the *p* value between food group and gut microorganism (Blue indicates *p* value <0.05, grey indicates *p* value >0.05).

Based on the significant seasonal differences of natural food types and gut microbiota in golden snub-nosed monkeys, we conducted a correlation analysis between food types and OTUs in four seasons. The results demonstrated that *Dendrobenthamia japonica* and *Zea mays* were correlated with gut microbiota in spring (Mantel test, *p* < 0.05, Figure 6A), and *Callicarpa nudiflora* was correlated with gut microbiota in summer (mantel test, *p* < 0.05, Figure 6B). In autumn and winter, there was no correlation between food types and gut microbiota (Figures 6C,D).

### 3.6. WGCNA on the hub OTUs

**Figure 7:**
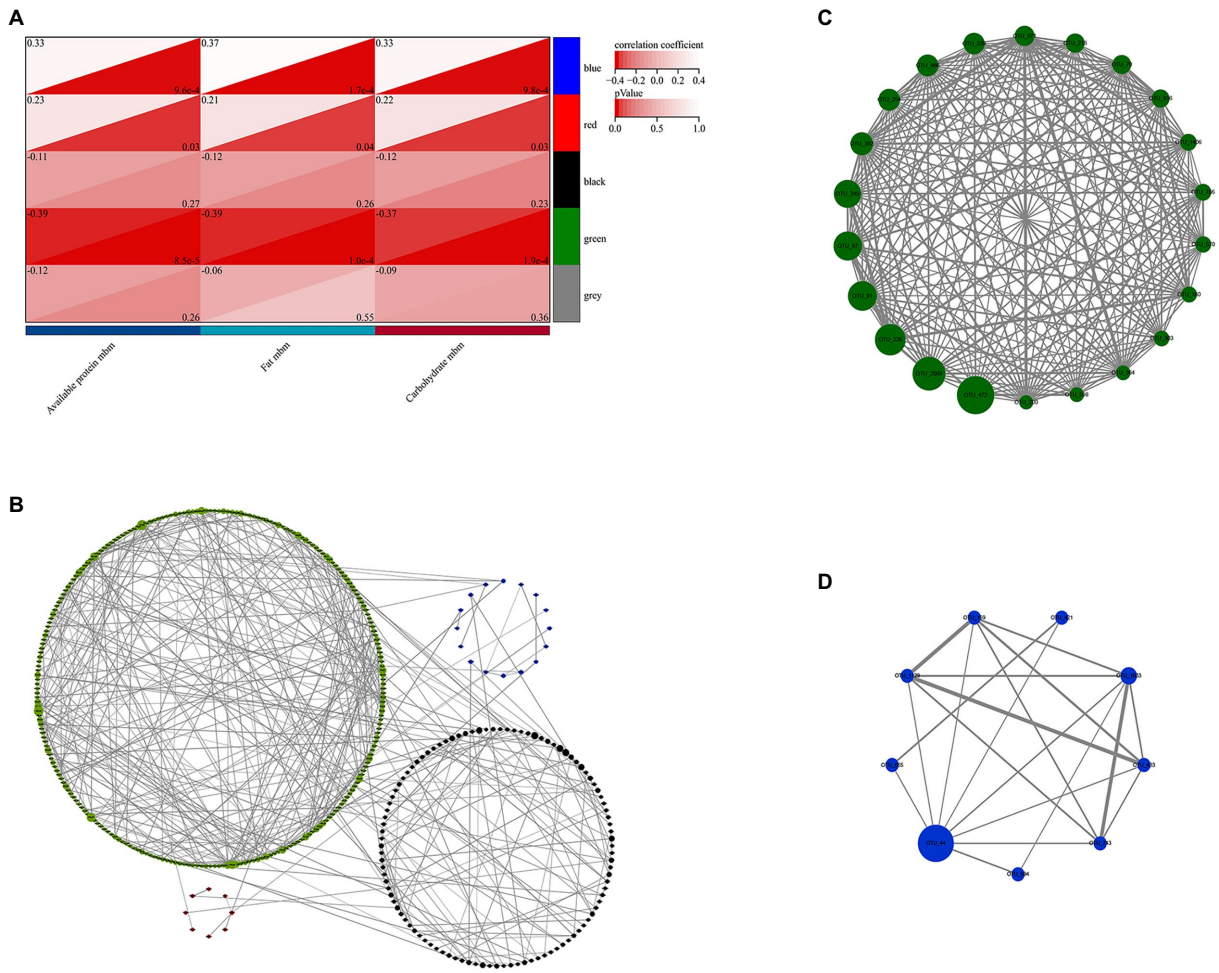
Identification of key module and hub OTUs based on WGCNA. **(A)** Correlation between module eigenvalues and traits of golden snub-nosed monkey. Depth of color corresponds to depth of correlation and p value of each module. **(B)** Network graph of the hub OTUs. Each node represented the OTUs whose betweenness centrality value was in the top 10%, and its color represented the corresponding module, the size of each node represented the betweenness centrality value, the size of each line thickness represented the weight value between nodes (OTUs). **(C)** Visualization of full weighted networks of 22 candidate hub OTUs in green module associated with three different nutrients (fat, protein, and carbohydrate). **(D)** Visualization of full weighted networks of 9 candidate hub OTUs in blue module associated with fat.

The present study used 3,638 OTUs for weighted gene co-expression network analysis (WGCNA). OTUs that exist over half of the sample in each season were selected and the network was constructed in one step. To define the adjacency matrix based on the criterion of approximate scale-free topology (Supplementary Figure S5), the network type was set as sign and the soft threshold parameter set to 10 with a minimum module size of 30 and the module detection sensitivity DeepSplit of 3. Modules that are correlated above 0.75 would be merged (Supplementary Figure S6). The clustering results showed that a total of 814 OTUs were parsed into 5 different modules. The gray module refers to ones that were not classified. The correlation between module eigenvalue and trait was calculated. The module-trait relationship heatmap demonstrated the correlation coefficient between module eigenvalues and traits. The green module refers to OTUs that were significantly correlated with three macronutrients (fat, carbohydrate, and available protein) at the same time, and the blue module was significant correlation with fat (*p* < 0.05, Figure 7A).

In addition, OTUs with betweenness centrality at top 10% in WGCNA were selected and the network graph was constructed in Cytoscape. The results showed that green module took the largest proportion and had richer network relationships (Figure 7B). Therefore, the green and blue modules were selected for hub gene analysis. We calculated the correlation between the module membership (MM) and the genes significance (GS) and nutritional traits. It was found that the relationship between MM and GS for these modules was relatively strong, particularly for those in the green module (*r* > 0.4, *p* < 0.05, Supplementary Figure S7). We also identified 22 candidate hub OTUs that are correlated with fat, carbohydrate and available protein in the green module, and 9 candidate hub OTUs that are correlated with fat in the blue module (threshold values of MM > 0.6 and GS < 0.1, respectively). The network graphs were constructed accordingly (Figures 7C,D). OTU_472, OTU_2009, OTU_226, OTU_81, OTU_67, and OTU_349 in the green module and OTU_44 in the blue module were found to be the most important hub OTUs. They belonged to the family *Ruminococcaceae* (OTU_472 and OTU_81), *Lachnospiraceae* (OTU_2009, OTU_226, and OTU_349) in the order *Clostridiales* and family *Muribaculaceae* (OTU_67), and *Prevotellaceae* (OTU_44) in the order *Bacteroidales*, respectively.

## 4. Discussion

Our results reveal that there are remarkable seasonal differences in both food items and macronutrients intake from food for wild golden snub-nosed monkeys. However, seasonal feeding strategies cannot fully explain the composition and fluctuation of their gut microbiota. On the contrary, the changes in carbohydrates, fats, and available proteins present similar trends with the changes of gut microbiota. Our findings suggest that the key factor that shapes the composition and function of wild golden snub-nosed monkeys’ gut microbiota is macronutrient intakes rather than food types. This differs from the view that diverse gut microbiotas in wild mammals are the result of the seasonal changes in dietary habits or food types in the previous studies.

### 4.1. The effects of nutrients on gut microbial composition

Previous studies suggest that seasonal changes in the composition and function of mammalian gut microbiota may be related to seasonal changes in the host’s diet. A study on wild blue sheep (*Pseudois nayaur*) found that changes in the composition of the animals’ gut microbiota is due to seasonal shifts in dietary habits (Zhu et al., 2020). Giant panda (*Ailuropoda melanoleuca*) gut microbes would produce more single-chain fatty acids during the shoot-eating season compared to the leaf-eating season (Huang et al., 2022). For wild geladas (*Theropithecus gelada*), the gut microbes in the rainier periods are mainly cellulolytic or fermentative bacterial that specialized in digestion grass, while during dry periods the gut is dominated by bacteria that break down starches found in underground plant parts (Baniel et al., 2021).

Meanwhile, many studies also suggest that the community structure and function of gut microbiota are influenced by the macronutrients consumed by host. In human guts, *Bacteroides* will be the dominant microbes when diets are rich in proteins and fats, while *Prevotella* is central to the diet rich in carbohydrates (Devkota et al., 2012; Henao-Mejia et al., 2012). Shifts in nutrient intakes have also been found to lead to changes of gut microbial composition in captive mammals. For example, high-fat diet with a high cholesterol intake resulted in dysbiosis of gut microbes and downregulation of microbial tryptophan metabolism in mice (Zhang et al., 2021a). Increasing different types of carbohydrates in the feed could lead to changes in the abundance of gut microbes in pigs (Lyu et al., 2020; Wang et al., 2021). Gut microbes in dogs and cats regulated their growth, reproduction, and homeostasis *per se* by breaking down nutrients that were not digestible by host digestive enzymes (Oh et al., 2021).

The analysis of the composition of gut microbiota in this study shows that both alpha-diversity and beta-diversity exhibited differences in richness and diversity in different seasons. *Firmicutes* was the most dominant phylum in all seasons, followed by *Bacteroidetes* and *Proteobacteria*. The results also indicate that *Firmicutes* can interact with other gut microbes to influence the absorption of nutrients. The previous study reveals that a high proportion of *Firmicutes* means that the host is able to get more energy from the food (Ley et al., 2006). When metabolic disorder produces dysbiosis that disturb the stability of the gut microbes, it is usually accompanied by an increase in *Proteobacteria* (Shin et al., 2015). In our study, golden snub-nosed monkeys have the least nutrient intakes in spring, and the gut microbiota show high abundance in *Firmicutes* and low abundance in *Proteobacteria*. This reflects that gut microbiota of host would be more stable in spring and the host could absorb more energy from low nutrient intakes to sustain life activities. Based on the above findings at the phylum level, we speculate that these gut microbes flourish to compensate for the low nutrient intakes in spring through microbial action to maximize energy utilization.

At the order level, samples in spring show an increase in *Methanobacteriales* and a decrease in *Aeromonadales*. Methanogens can reduce intestinal gas accumulation (Kengen et al., 1994) and maintain an anaerobic environment in the hindgut, facilitate the metabolic of polysaccharides, and improve the utilization efficiency of energy (Samuel and Gordon, 2006; Samuel et al., 2007). And more our study finds that the abundance of *Aeromonadales* is much lower in spring than in other seasons, while the increased abundance of *Aeromonadales* has been verified to be the reason for intestinal inflammation. *Aeromonadales*-related lipopolysaccharides disrupt the intestinal mucosal barrier and cause the increase of intestinal permeability, thereby causing inflammation (Zhang et al., 2021b).

At the genus level, there is an increase in the genus *Methanosphaera*, *Methanobrevibacter*, *Shuttleworthia*, *Ruminococcaceae_UCG-014*, *Treponema_2*, *Ruminiclostridium*, and *Ruminococcaceae_NK4A214_group* in spring when proteins, fats, and carbohydrates are consumed at the lowest amount. The methanogenic bacteria *Methanosphaera*, *Methanobrevibacter*, and *Shuttleworthia* can convert hydrogen and formate into methane. When enriched simultaneously with efficient hydrogen-producing bacteria such as *Christensenellaceae_R-7_group* (Morotomi et al., 2012), they are able to work synergistically to improve the efficiency of gut fermentation of starch and other polysaccharides (Samuel and Gordon, 2006). A large number of methanogenic bacteria can increase the calories obtained from food and enhance the absorption and utilization of nutrients (Mizrahi et al., 2021). They also promote the production of short-chain fatty acids by other fermenting bacteria and stimulate the production of fats (Zhang et al., 2009; Basseri et al., 2012). *Ruminococcaceae_UCG-014* can produce butyrate, an important energy source for colon cells. In the meantime, they can increase short-chain fatty acids and affect host appetite and satiety through different mechanisms, delaying gastric emptying and thus energy absorption (Canfora et al., 2015; Guo et al., 2022). *Ruminiclostridium* is positively correlated with acetate content in the cecum, providing more energy to the host (Zhang et al., 2021c). As to autumn when diets are high-carbohydrate and high-fat, *Prevotella*, *Phascolarctobacterium*, and *Lactobacillus* were the dominant genus. *Prevotella* is capable of breaking down non-cellulosic polysaccharides and pectins (Flint et al., 2012). Both *Phascolarctobacterium* and *Lactobacillus* are probiotics that can break down fats (Lv et al., 2019; Rodrigues et al., 2021). Although our results show that the change in gut microbiota from spring to autumn is not due to a change in diet structure. However, there are diverse polysaccharides and fats that could be potentially utilized in autumn. Therefore, we suggest that differences in nutrient intakes may be a significant factor that shaped the composition of gut microbial communities during animal growth and development.

### 4.2. The effects of nutrients on gut microbial function

The analysis for gene function prediction based on KEGG database shows that the gut microbiota in wild golden snub-nosed monkeys mainly take part in metabolism and synthesis of lipid, carbohydrate, protein, amino acids, and other secondary metabolites. We should point out that most of the functional genes in the metagenome in this study appear to be involved in carbohydrate metabolism, but these pathways are not enriched in any seasonal grouping. This result may be due to the fact that we fed the monkeys equal amounts of maize throughout the year, which maintained their energy provided by large amounts of carbohydrates at a relatively constant level. Therefore, the gut microbes also responded stably to the degradation of carbohydrate.

The macronutrients are found significantly different both between spring and summer groups and between summer and winter groups. Also, there is a significant difference in fat intakes between spring and autumn groups. We found that 71, 228, and 210 differentially expressed genes between summer vs. winter group, spring vs. summer group, and spring vs. autumn group and were significantly enriched in 16, 20, and 21 KEGG pathways, respectively. Six of these pathways are important for the response to changes in the nutrients intake, including protein digestion and absorption (ko04974), fatty acid elongation (ko00062), arachidonic acid metabolism (ko00590), linoleic acid metabolism (ko00591), alpha-linolenic acid metabolism (ko00592), and bile secretion (ko04976) (Figure 6). In fact, there are studies reporting that these pathways play an important role in physiological activities of the host. For instance, bile acid is an amphiphilic molecule with strong surface activity (Yang et al., 2020), which can emulsify fat into chylomicrons to increase the contact area between lipase and fat, and facilitates fat digestion and reduces autologous fat catabolism (Velazquez-Villegas et al., 2018; Hu et al., 2019). Alpha-linolenic acid, linoleic acid, and arachidonic acid are essential fatty acids that animals cannot be synthesized by the body and must come from food (Di Pasquale, 2009; Martin et al., 2016). These results indicate that seasonal differences in these pathways may mainly be due to the differences in nutrient intakes.

Noticeably, in season groups with significant differences in nutrient intakes, besides the pathway associated with macronutrient metabolism, we observed enrichment of multiple secondary metabolite biosynthesis pathways such as Phenylpropanoid biosynthesis, Flavone and flavonol biosynthesis, Flavonoid biosynthesis, and Diarylheptanoid and gingerol biosynthesis. As signals to gut microbes, microbial diet-based metabolites or small molecules are key mediators that affect physiological processes in the host (Koh et al., 2016). They can activate or inhibit endogenous signaling pathways, or act as a source of nutrients for host cells (Sonnenburg and Bäckhed, 2016). The biosynthesis of phenylpropane begins with the shikimate pathway, which initially breaks down glucose by the combined action of the glycolysis and pentose phosphate pathway to produce phosphoenolpyruvate and erythrose-4-phosphate of the synthetic initiation metabolite 3-deoxy-D-arabino-heptulosonic acid 7-phosphate (DAHP) (Chen et al., 2016). Flavone, flavonol, and flavonoid metabolites all appear as intermediates in phenylpropanoid biosynthesis pathway (Dong and Lin, 2021). In addition, key enzymes for the synthesis of resveratrol (stilbenoid), diarylheptanoid, and gingerol are also the central nodes of the phenylpropane pathway (Yin et al., 2022). Therefore, the enrichment of these pathways may be related to the reutilization of host-ingested carbohydrates by gut microbes. This implies that the intake of macronutrients exceeds the digestibility during seasons when foods were abundant. The macronutrients escape primary digestion and become a substrate for microbial metabolism to produce fermentation by-products and affect host physiological health.

The results of WGCNA analysis indicate that the green module has the highest correlation with fat, protein, and carbohydrate, and it also has the most complex network relationships. Twenty out of 22 candidate members of hub OTUs belong to *Clostridiales* and two belong to *Bacteroidales*. A total of five families have been annotated, namely *Ruminococcaceae*, *Lachnospiraceae*, *Muribaculaceae*, *Peptococcaceae*, and *family_XIII*. Among them, candidate members of *Ruminococcaceae* (OTU_472, OTU_81, OTU_339, OTU_570, and OTU_598) show consistent inter-seasonal trends with the energy provided by carbohydrates in natural foods in terms of their abundance, this indicates that these OTUs also have the lowest abundance in spring when carbohydrate intake was lowest. Previous studies have proved that *Ruminococcaceae* can degrade a variety of polysaccharides and dietary fibers. They are also the producers of short-chain fatty acids (SCFAs) (Scott et al., 2008; Louis and Flint, 2009; Hooda et al., 2012). Our assumptions about the relationship between nutrients and gut microbes are consistent with these findings. OTU_472, OTU_2009, OTU_226, OTU_81, OTU_67, and OTU_349 have largest fluctuation with changes in nutrients intakes. These 6 hub OTUs were annotated to *Ruminococcaceae*, *Lachnospiraceae*, and *Muribaculaceae*. They were at the core of the green module and largely affect the network structure of the co-occurrence bacterial taxa network pf the green module. This could be used as an important indicator to assess the gut nutrient absorption of the golden snub-nosed monkeys.

### 4.3. The evolution of host adaptation

Based on the present finding that macronutrients are responsible for the changes in golden snub-nosed monkeys’ gut microbiota, we consider this is an important mechanism that helps them survive and increase fitness. This can be inferred from the great number of gut microbes and metabolic pathways annotated in the study. In our study, the gut microbiotas of golden snub-nosed monkeys were annotated to 38 phyla, 140 orders, 352 genera, and 395 metabolic pathways. We refer to previous studies that found golden snub-nosed monkeys have more types of gut microbes and metabolic pathways compared to mammals with relatively homogeneous or food-specific diets such as red pandas (*Ailurus fulgens*) (Kong et al., 2014), koalas (*Phascolarctos cinereus*) (Barker et al., 2013), amur tigers (*Panthera tigris altaica*) (Ning et al., 2020), and musk deers (*Moschus chrysogaster*) (Sun et al., 2019). This is unlikely due to the sequencing depth because the Good’s coverage of each bacterial community was >97%. Golden snub-nosed monkeys rely on microbiota functions to obtain sufficient nutrients from foods to cope with the harsh living conditions and variable food types (Liu et al., 2022). We infer that the gut microbiota of golden snub-nosed monkeys has gradually become more diverse and complex during their co-evolution with their hosts to stabilize the host nutrient intakes under seasonal shifts of the diet. The gut microbiota helps the host adapt to broader dietary by enabling the host to digest multiple food types and obtain sufficient nutrients to meet its survival needs.

## 5. Conclusion

Golden snub-nosed monkeys exist significant difference in food consumed and nutrients intake among seasons that the three macronutrients intake showed a consistent trend that they are higher in summer and autumn and lower in spring and winter. We found seasonal dietary differences caused the macronutrient variation is the main reason for seasonal shifts of gut microbiota. Particularly, phyla *Firmicutes*, *Bacteroidetes*, and *Proteobacteria* are significantly dominant in all samples, but the ratio of Firmicutes to Bacteroidetes was correspondingly highest in spring when nutrient intakes were lowest per metabolic body weight. The dominant genera also showed the same seasonal trends: Methanogens and *Ruminococcus*, which promote nutrient intake efficiency increased in spring when nutrient intakes were lowest. In autumn when high-carbohydrate and high-fat diets were consumed, *Prevotella* that digest complex polysaccharides had a high abundance. These results demonstrated that gut microbes through microbial metabolic functions help the host to compensate for the insufficient macronutrients intake.

## Data availability statement

The data presented in the study are deposited in the https://www.ncbi.nlm.nih.gov/bioproject/929077, repository accession number PRJNA929077.

## Ethics statement

The animal study was reviewed and approved by **t**he Ethics Committee of Northwest University.

## Author contributions

SG conceived the project idea and designed the study. HF and SH collected the samples and performed the experiments. YL, YY, and ZW performed the bioinformatic analysis of the sequencing data. YL and YY wrote the manuscript and analyzed and interpreted the data. YL, YY, SJ, and SG edited the manuscript. All authors read and approved the final manuscript.

## Funding

This project was sponsored by the Major International Joint Research Program of Natural Science Foundation of China (NSFC) (32220103002), research programs of NSFC (31872247), the Strategic Priority Research Program of the Chinese Academy of Sciences (XDB 31020302), and the Qinghai Province High-level Innovative “Thousand Talents” Program.

## Conflict of interest

Author GD was employed by Guangdong Chimelong Group Co., Ltd.

The remaining authors declare that the research was conducted in the absence of any commercial or financial relationships that could be construed as a potential conflict of interest.

## Publisher’s note

All claims expressed in this article are solely those of the authors and do not necessarily represent those of their affiliated organizations, or those of the publisher, the editors and the reviewers. Any product that may be evaluated in this article, or claim that may be made by its manufacturer, is not guaranteed or endorsed by the publisher.
