# Supplementary material for: Does diet or macronutrients intake drive the structure and function of gut microbiota?
Source: Front Microbiol. 2023 Feb 13;14:1126189. doi: 10.3389/fmicb.2023.1126189 (PMC9970161; doi:10.3389/fmicb.2023.1126189)
Supplement: Supplementary file 1 [file Data_Sheet_1.docx]

Supplementary Material

## Supplementary Figures


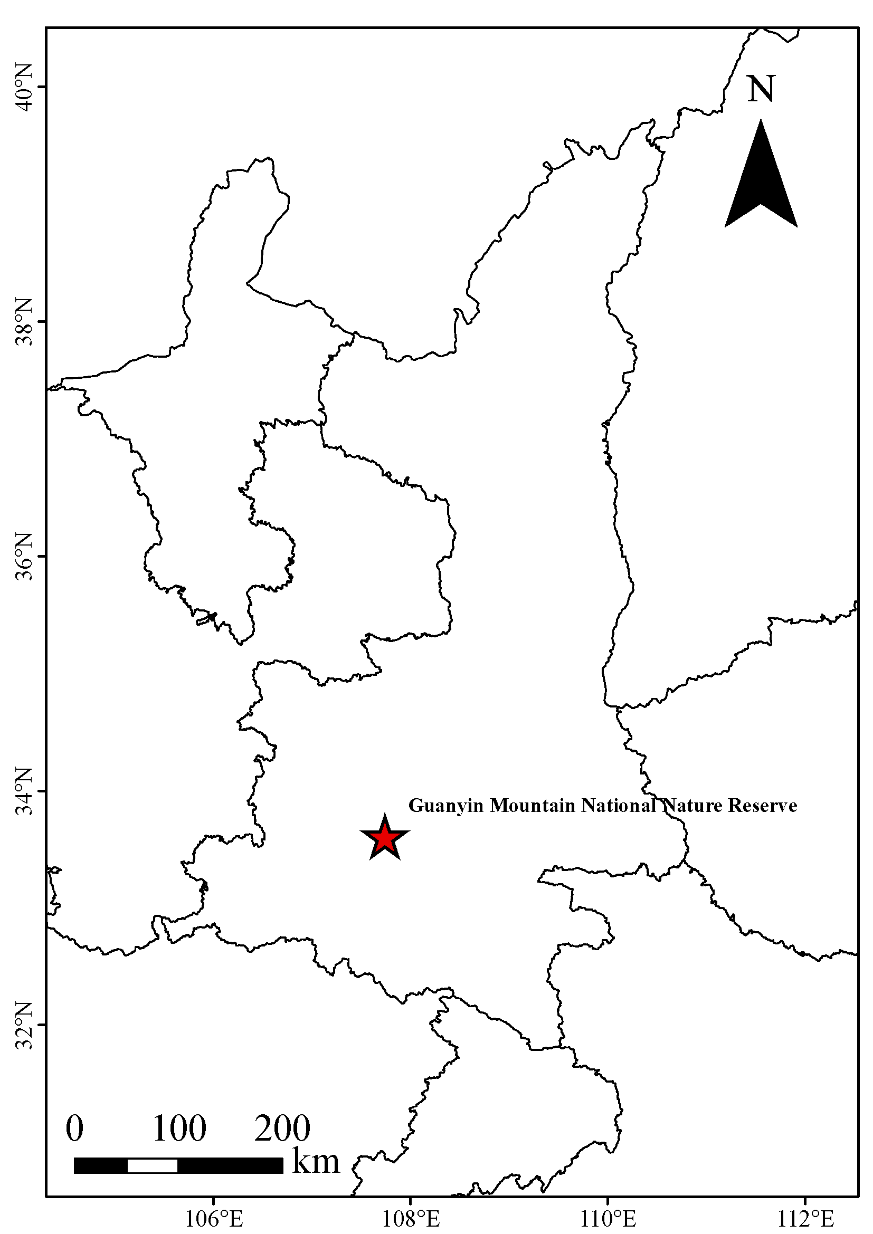


**Supplementary Figure 1.** Location of study site.

**
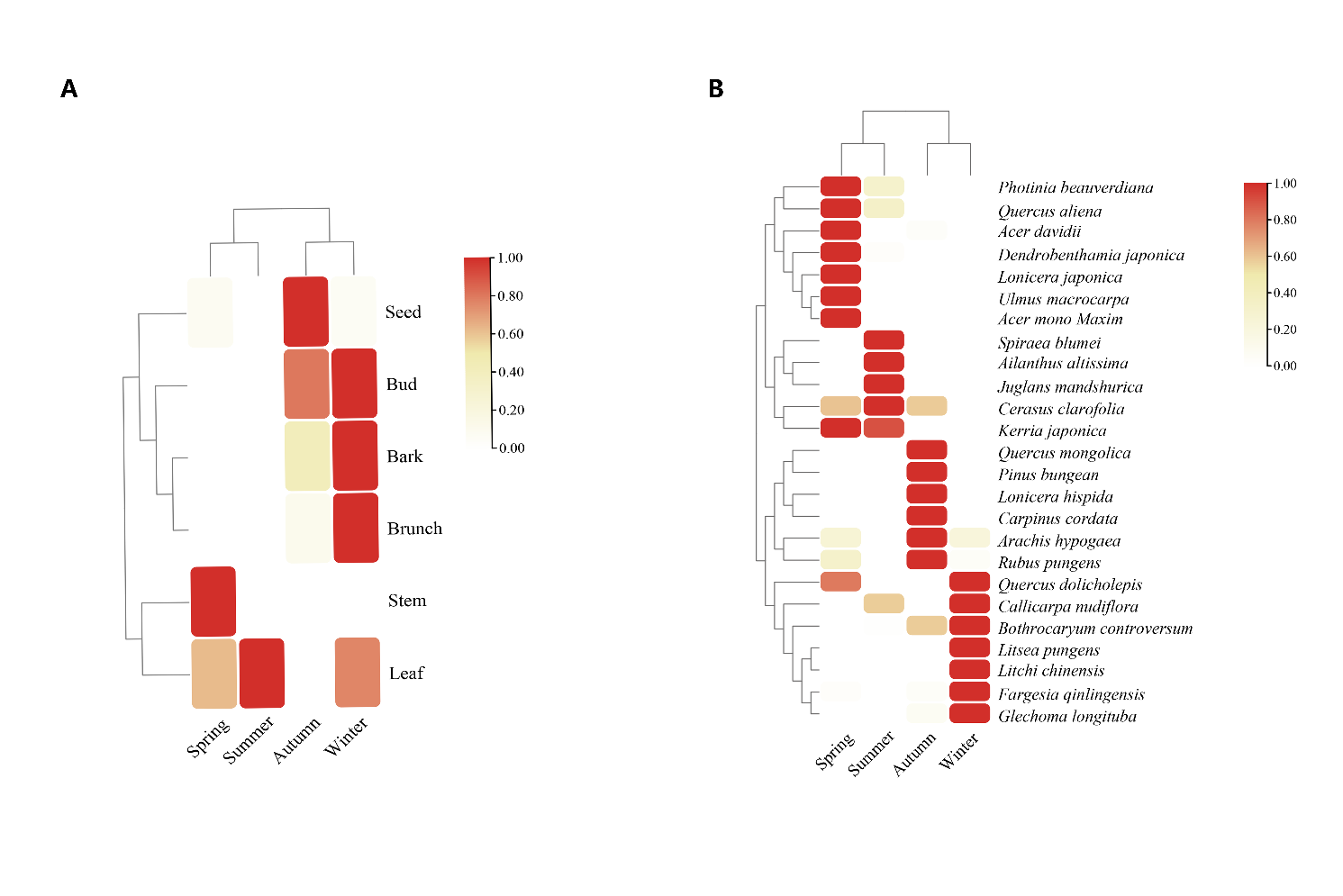
**

**Supplementary Figure 2.** Heat map of the food species **(A)** and plant parts consumed **(B)** of golden snub-nosed monkeys in four seasons. The deeper the red, the higher the proportion of this species taken in this season compared to other seasons.

**
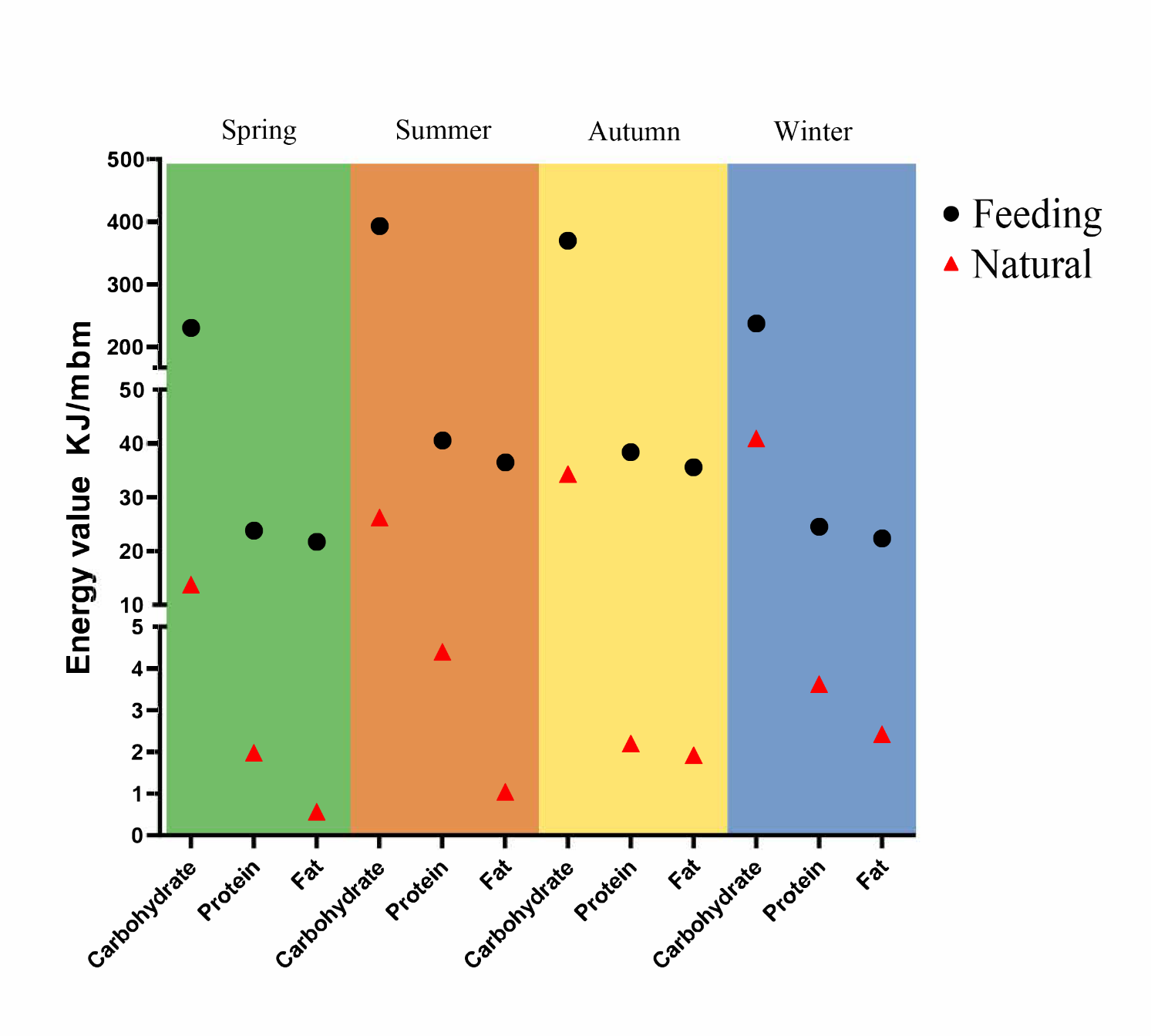
**

**Supplementary Figure 3.** Scatterplot of energy intake per metabolic body weight from macronutrients in artificial and natural foods over four seasons, with black dots for artificial foods and red triangles for natural foods.


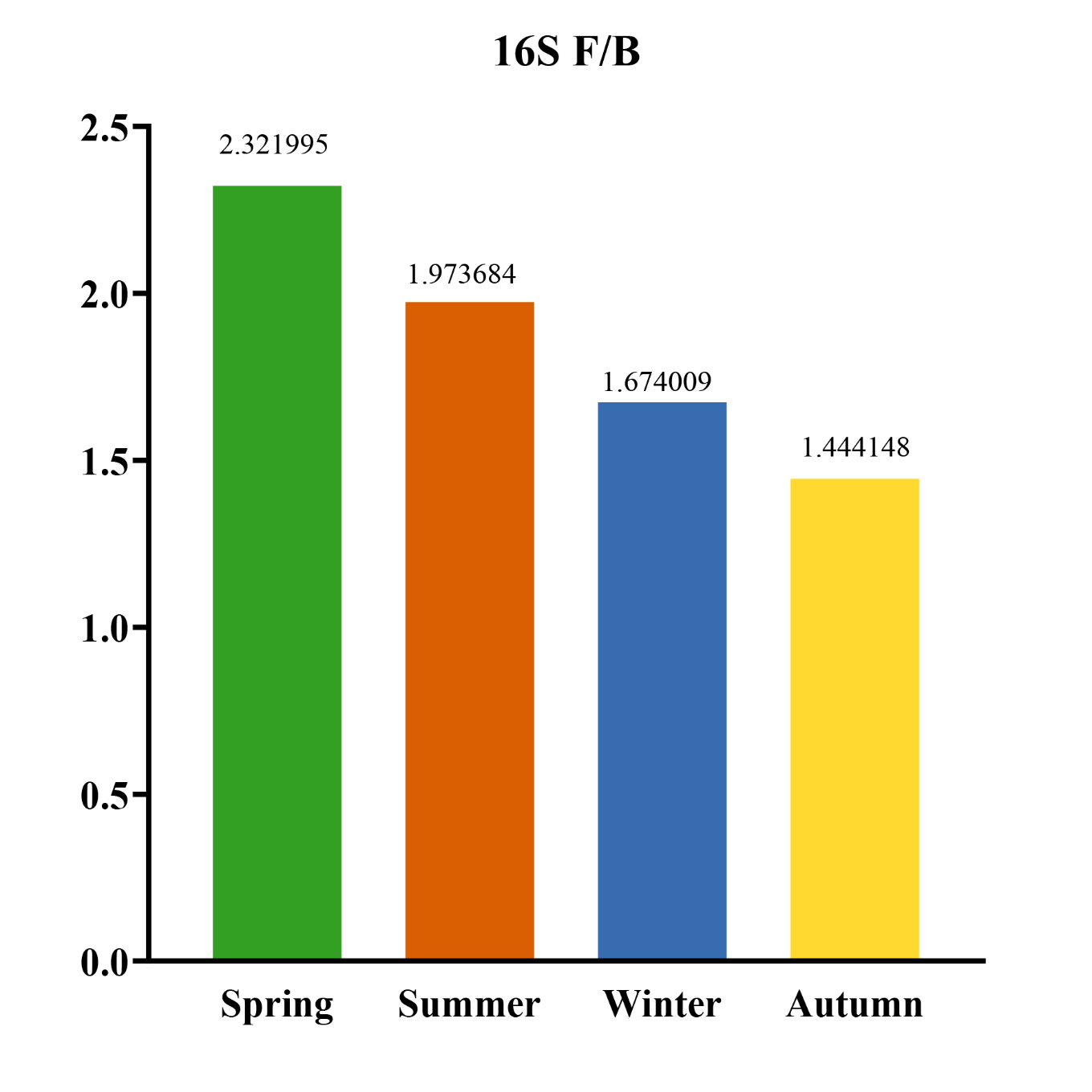


**Supplementary Figure 4.** Analysis of seasonal differences in the ratio of Firmicutes to Bacteroidetes (F/B ratio).


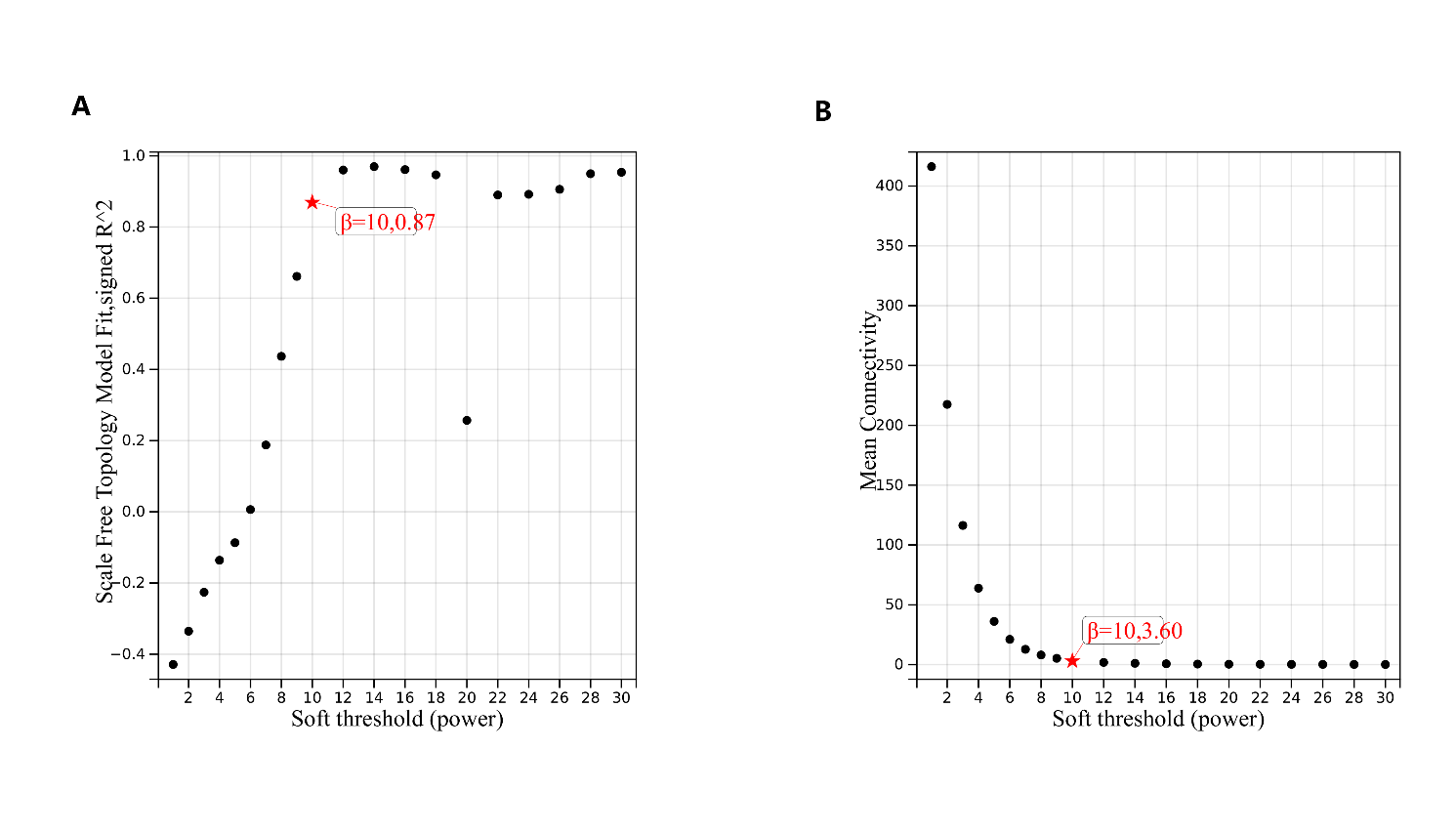


**Supplementary Figure 5.** Analysis of network topology for various soft-thresholding powers. The top panel shows the scale-free fit index (y-axis) as a function of the soft-thresholding power (x-axis). The bottom panel displays the mean connectivity (degree, y-axis) as a function of the soft-thresholding power (x-axis). Network topology for 1 to 20 soft-thresholding powers. Numbers in the plots indicate the corresponding soft-thresholding powers. The red line indicates scale-free topology fit index at 0.87 **(A)** as well as blue line indicate mean connectivity at 3.60 **(B)**. The approximate scale-free topology can be attained at the soft-thresholding power of 10.


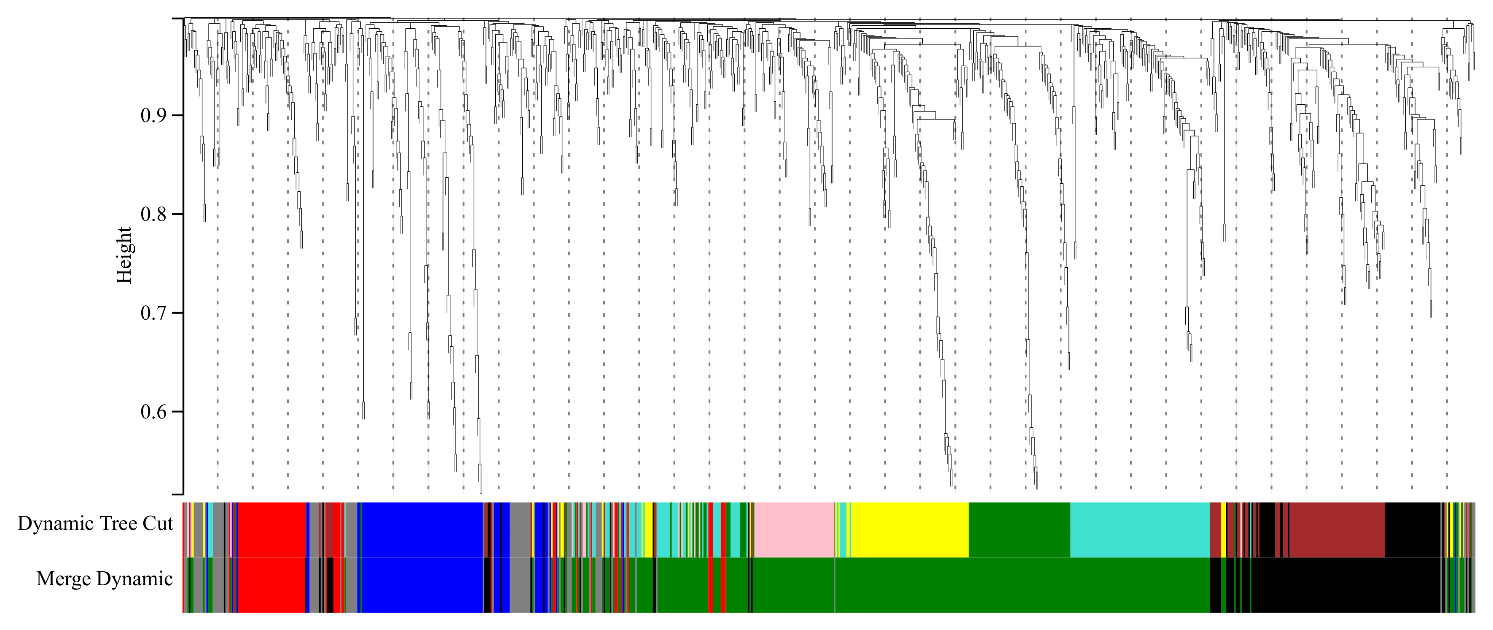


**Supplementary Figure 6.** Clustering dendrogram of bacterial taxa, with dissimilarity based on topological overlap, together with assigned module colors.


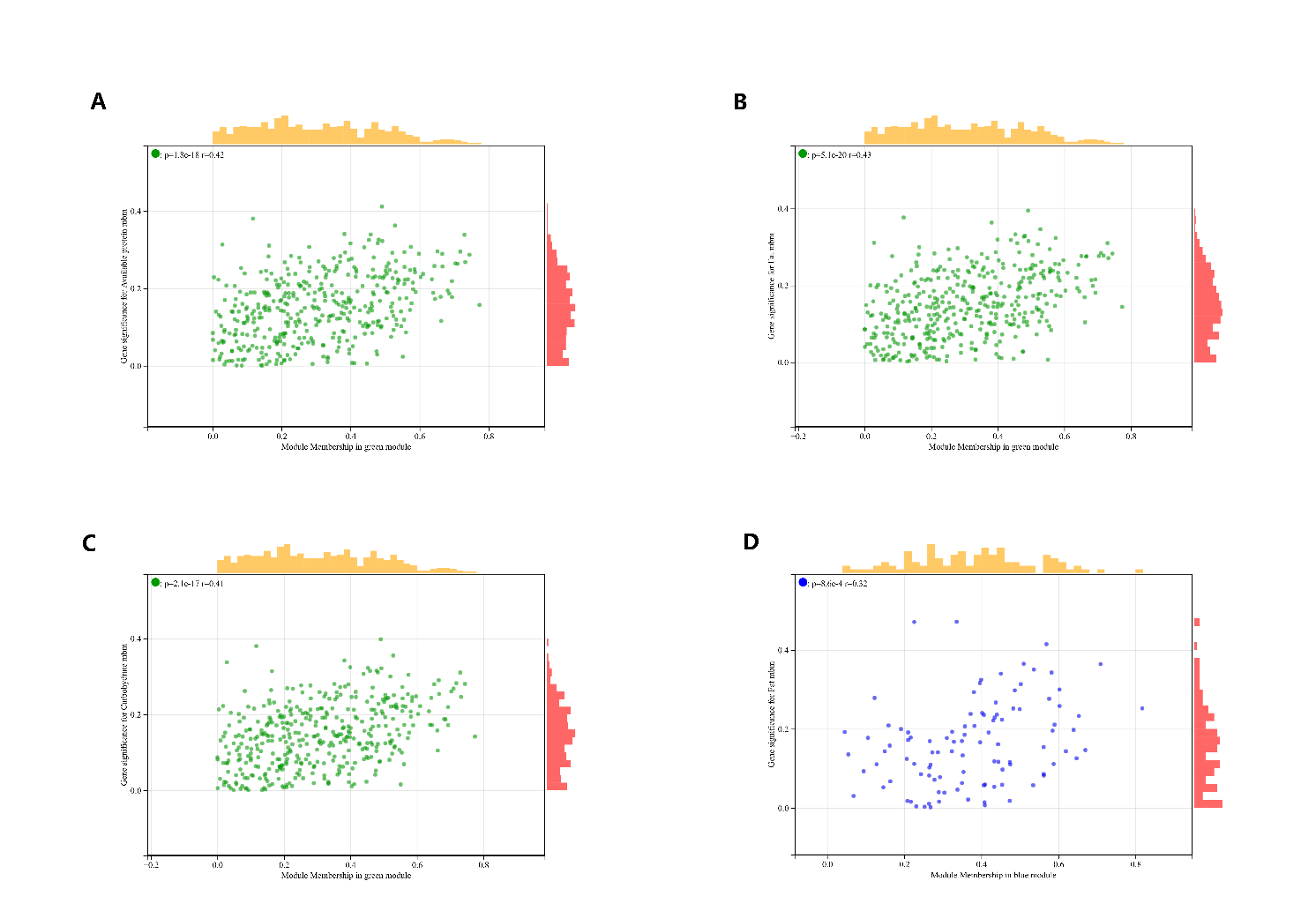


**Supplementary Figure 7.** Scatter plot of GS and MM correlations. **(A)** The p-value of available protein and green model is 1.8e-18, and the R-value is 0.42. **(B)** The p-value of fat and green model is 5.1e-20, and the R-value is 0.43. **(C)** The p-value of carbohydrate and green model is 2.1e-17, and the R-value is 0.41. **(D)** The p-value of fat and blue model is 8.6e-4, and the R-value is 0.32.
